# GIS-based framework for artificial aquifer recharge to secure sustainable strategic water reserves in Qatar arid environment peninsula

**DOI:** 10.1038/s41598-021-97593-w

**Published:** 2021-09-14

**Authors:** Yasir Elginaid Mohieldeen, Elnaiem Ali Elobaid, Rifaat Abdalla

**Affiliations:** 1 Qatar Environment and Energy Research Institute (QEERI), Hamad Bin Khalifa University (HBKU), Qatar Foundation, Doha, Qatar; 2 Environmental Science Center (ESC), Qatar University, H10-Zone 3-B113, P.O. Box 2713, Doha, Qatar; 3 Department of Earth Sciences, College of Science, Sultan Qaboos University, Muscat, Oman

**Keywords:** Environmental sciences, Hydrology

## Abstract

This study proposes a large-scale artificial aquifer recharge plan to increase the strategic water reserve to cope with future emergencies. The main aim of the plan is to restore groundwater levels to those of the 1980s through artificial recharge. Desalinated water or highly treated municipal sewage effluent could be artificially recharged into the aquifer to recharge it. Potentiometric surface of aquifers and Geographic Information Systems (GIS) analysis were used to assess change in the groundwater levels between 1980 and 2009. Zones that have experienced considerable decline in groundwater levels from their former “natural” status—when the aquifers were barely exploited, were identified. These zones are considered optimum recharge sites as they could provide ‘natural’ ground storage chosen by nature. Therefore, working with nature (not against it) by re-filling these natural spaces is the optimum approach. The artificial recharge of the main and principal upper aquifer in Qatar (Rus and Um er Radhuma) is targeted and recommended. It is estimated that up to 182.8 Million Cubic Meter (mcm) could be recharged and stored in these proposed zones, to increase the strategic water reserve of the country. This increase would sustain supplies of high quality for up to three months if consumption is maintained at the 2018 level. Moreover, this additional reserve could last for over one year, if emergency measures were put in place—in case of serious water-shortages, and disaster preparedness, for example by reducing the per capita consumption to the global average per capita consumption.

## Introduction

Qatar strategic water reserves is enough for only seven days, according to official statistics, as most of the Gulf Council Cooperation (GCC) countries. With very low annual rainfall desertic hyper-arid environment, no surface water, and depleted groundwater, the country is considered as one of the most water scarce countries in the world. The country has been relying strongly on desalinated water, for domestic consumption, from the Arabian/Persian Gulf (simply referred to and hereafter as the Gulf) since the early 2000s. This reliance on desalination makes the country very vulnerable to disasters in the Gulf that could prevent water intake to the desalination plants. Natural disasters such as red-tide outbreak, and man-made disasters such as oil-spills are examples of these disasters that could stop desalination plants in the Gulf for days, possibly months. This vulnerability is exacerbated by the fact that strategic water reserve in the State of Qatar is enough for only seven days. Seven days is not enough time for authorities to recover from such disasters. For instance, the 2010 oil spill that took place in the Gulf of Mexico, took more than one month to clear the oil before the water could be utilized. Another example is the 2008 red-tide outbreak in the Gulf that lasted for over eight months and forced the closure of the desalination plants in Qatar and other Gulf countries for weeks. In addition, the fossil fresh groundwater resources in the country have been exploited heavily during the past few decades, mainly for agricultural purposes. The continuous over-abstraction has negatively affected groundwater quantity and quality, as consequence of seawater intrusion and the upward leakage of brackish water. Qatar’s current groundwater would be unusable for human consumption in the event of water shortage emergency caused by disasters in the Gulf or unexpected failure of the desalination plants. With very low annual rainfall with an average of about 80 mm/year and high rates of evaporation with average 11.5 mm/day^-^ in summer and 2.5 mm/ day in winter season, Qatar faces severe water scarcity. The average rainwater endowment of the country, in 2014, was less than 29 cubic meters per year per capita (m^3^/year/ca), compared to the global average of 6000 m^3^/year/ca, and a water poverty line of 1000 m^3^/year/ca^1^. Nevertheless, the per capita water consumption of the country is among the highest in the world, reaching more than 800 L per capita per day, whereas the global average is about 160 L per capita per day^2,3^. This high per capita consumption in Qatar is induced by the rapid urbanization and improved living standards since the early 1980s.

The Qatar 30-year water masterplan initiated in 2009 includes major investment in desalination, water infrastructure and wastewater treatment^4^. Between 2010 and 2015, the value of water projects reached 5.47 billion USD^4^.

The high dependency on seawater desalination in water production puts Qatar at a high risk from major threats of natural pollution events such as red-tide and harmful algal blooms (HAB), and anthropogenic sources such as oil spill events in the Gulf that could disrupt water intake at the desalination plants. Furthermore, the current strategic water reserves in Qatar would only be sufficient for seven days^1,4^. This limited strategic storage capacity renders Qatar *vulnerable* to major man-made and natural disasters such as oil spills and red-tides respectively, that might interrupt the operation of the desalination plants or cause them to shut down for a considerable period of time.

Increasing the strategic water reserve in Qatar is a major concern and priority for the authorities in Qatar. The General Electricity and Water Corporation (Kahramaa) has conducted studies on how to address this issue. Adopting the national water saving program (TARSHEED); embarking on the construction of seven-day water mega scale storage tanks as part of its water strategic plan^1,4^; and artificial recharge of water attempts are examples of these efforts.

The geographical scope of the study is the State of Qatar, and its upper principal aquifer (Rus, and Umm er Radhuma), where changes in groundwater levels between 1980 and 2009 were investigated. Knowing the magnitude of these changes and their spatial distribution is critical to any aquifer recharge plans. The main objective is to determine what is needed to replenish/restore the depleted aquifers to their 1980s levels (a time when the aquifer was hardly used) and use stored water as a natural strategic water reserve to meet water supply emergencies resulting from natural or man-made disasters. The suitability of the proposed zones was verified with hydrological, hydrogeological geotechnical experimental fieldwork and tests obtained from a study by Streetly et al. (1998), since their test sites are in close proximity to the zones proposed in this study, and therefore representative of these zones. Soil porosity and other geotechnical parameters of these test sites have been used for water volume calculations.

The study is structured as follows: First, a brief overview of the water resources in Qatar is provided, along with an overview of the types of disaster that could interrupt water services in the country. Secondly, there follows a description of the data used in the analysis. Thirdly, the methodology adopted to identify potential groundwater recharge zones is discussed critically. Finally, there are concluding comments and recommendations.

## Groundwater conditions in Qatar

### Water supply and demand

**Figure 1:**
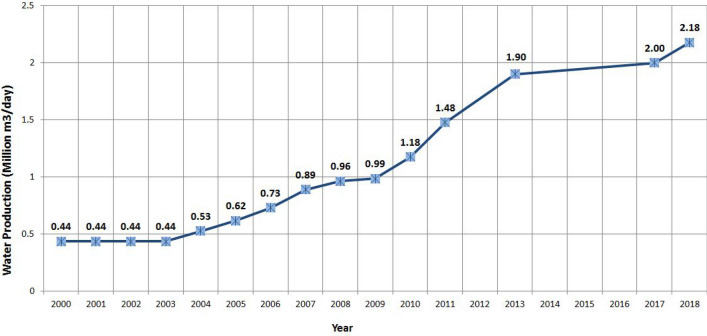
Water production in Qatar in mcm/day, 2000–2018^1,6^.

The groundwater Conditions in Qatar is very critical, whereas the environment of Qatar experiences a hyper-arid hot desert climate with very little rainfall. Water desalination from the Gulf is the main source of freshwater. In 2009, 99.9% of the domestic water, produced by the Qatari water company (Kahramaa), was desalinated while the rest was from groundwater^4^. Desalinated water production has increased fourfold, from 0.44 to 1.48 mcm/day, between 2003 and 2011 (Fig. 1)^5^. It was reported that seawater desalination reached 1.9 mcm/day in 2013^1^, and 2.18 mcm/day in 2018^6^.

### Problems and challenges

**Figure 2:**
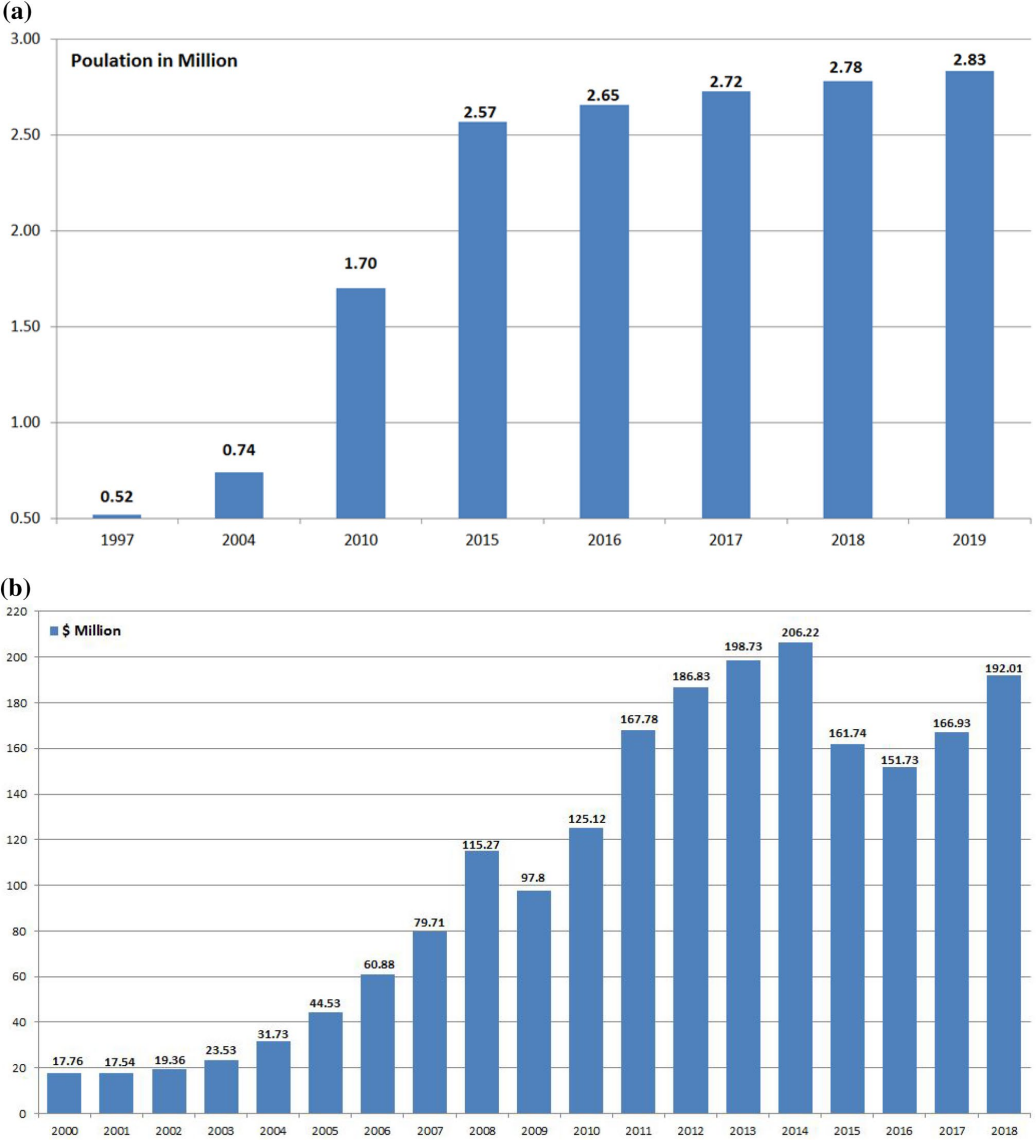
**(a)** Population growth in the State of Qatar during the period 1997–2019^1,7,8^. (**b)** Qatar GDP in billion USD, 2000–2018^9^.

There are some problems and challenges facing groundwater status in Qatar Peninsula, such as the rapid socio-economic transformation has taken place in Qatar, over the past few decades, has resulted in enormous pressure on natural resources especially groundwater resources. This pressure is attributed to the increased farming activities that the country has pursued to meet the increasing food demand of the rapidly rising population, and the demand associated with the improved standard of living. The population in Qatar has increased more than fivefold, from 0.52 Million to 2.83 Million between 1997 and 2019^1,7,8^ as shown in Fig. 2a. The Gross Domestic Product (GDP) of the country has also increased from 17.76 billion USD in 2000 to 192.01 billion USD in 2018^9^, as shown in Fig. 2b. Another reason for the increase in irrigated farming is a consequence of the high social status enjoyed by those who own farms and gardens.

**Figure 3:**
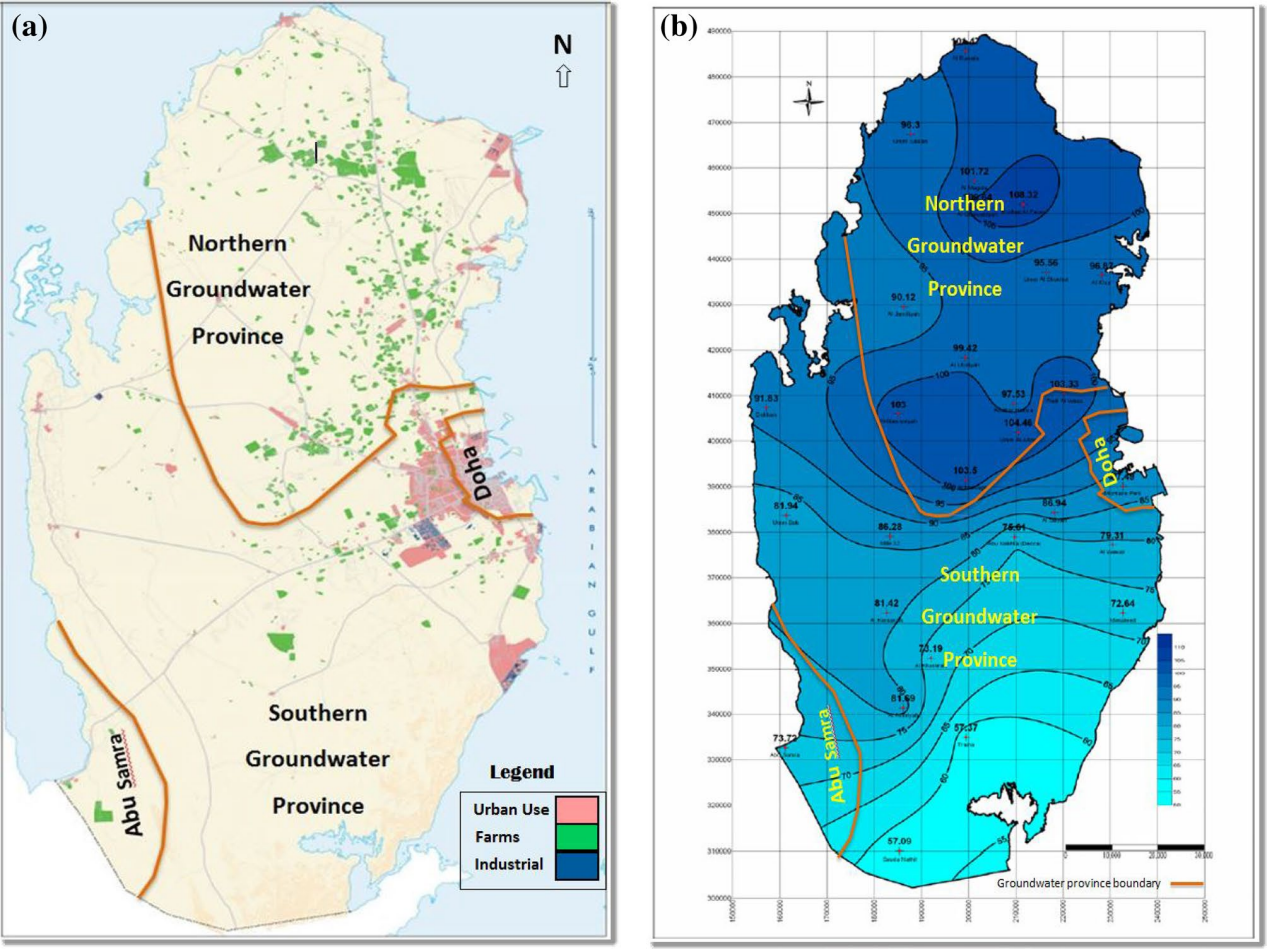
**(a)** Map of broad land use and farms distribution in the State of Qatar in 2012. (Source: Public Statistics Authority, Qatar http://www.psa.gov.qa)^33^. **(b)** Groundwater province boundaries and the distribution of annual average rainfall in mm during 1989 and 2007. (Source: Ministry of Environment, Technical Governmental Report Publically Available)^21^.

The spatial extent of groundwater aquifers in Qatar is sub-divided into two major groundwater provinces: the Northern and Southern provinces, and two small ones—namely the Doha and Abu Samra basins as in Fig. 3a. Most of the groundwater abstraction occurs in the farming area within the Northern groundwater province, as it receives more natural rainfall.

The Northern Province recharge from rainfall is 30% more than that of the Southern province, as shown in the rainfall map Fig. 3b.

**Figure 4:**
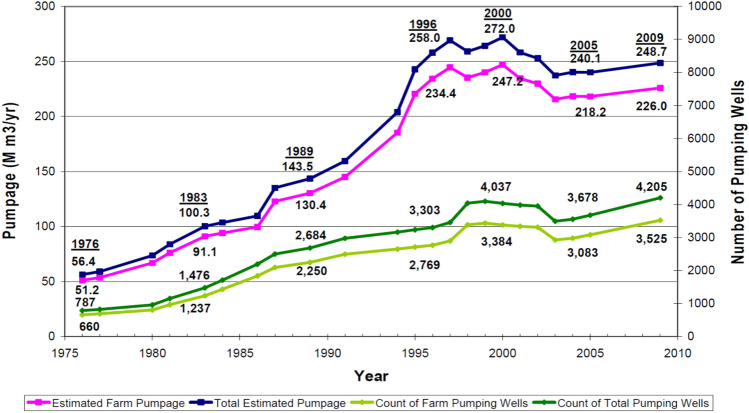
Groundwater abstraction & number of wells in the State of Qatar, 1975–2009^21^.

The abstraction of groundwater started to increase year by years since 1975. The continuous and rapid increase in the number of farms, along with increased number of wells inside these farms, shown in Fig. 4, caused depletion and deterioration of groundwater in-terms of quality and quantity.

### Risks and major threats

#### Oil spill

The fact that Qatar relies heavily on the water desalination from the Gulf makes the country very vulnerable to pollution events in the Gulf, since it is very shallow^10^, compared—for example—with the Gulf of Mexico. As a result, in the case of a major oil spill the concentration of oil in the water would be very high and would take a long time to clean up. The 2010 oil spill that took place in the Gulf of Mexico took thirty-six days to clean up using the advanced equipment available in the USA, despite the different and more resilient depth and other conditions compared to the Gulf. Oil spills are the major type of disaster that could interrupt water production from the Gulf. While the Gulf represents only 0.066% of the World’s waters, more than 40% of the daily oil tankers traffic of the World passes through it^11^, since the Gulf region is the leading world exporter. For instance, about 17 billion barrels of oil per day were transported through the Strait of Hormuz in 2011 and accounting for 20% of the World’s oil^12^. This dense traffic of tankers in the Gulf makes the probability of major oil spill disasters very high. Many small to intermediate scale oil spills from oil tankers have been detected and reported by different agencies using different detection techniques, including remote sensing.

#### Red-tide phenomena

Red-tide is formed by the growth of *Karenia brevis* HAB, which is a microscopic, single-celled, photosynthetic organism. In the last few decades red-tide outbreaks have become very frequent in the Gulf. For instance, major outbreaks in the Gulf occurred during the winter seasons of 2000, 2001, 2006, 2008–2009^13^, and 2010. The 2008–2009 red-tide event lasted for more than eight months, forcing the closure of desalination plants in Qatar and the region^14^. Barka desalination plant in Oman, for instance, had to cease operations for 55 days^15^. Ironically, the increased temperature of the waters, due to the disposal of hot brine from desalination plants in the Gulf, is one of main reasons of the outbreak of the red-tides in the Gulf. Many studies have shed light on this recently developed phenomenon in the Gulf and its impact on desalination plants^14,16–18^, and on the environmental impact of desalination plants on marine environment^19,20^.

## Materials and methods

### Potentiometric surface of aquifers

Limited groundwater data is one of the main impediments that hinder the study of aquifer development and modeling in the Middle East. For this study, the only accessible data were two potentiometric iso-maps for the years 1980 and the 2009 Ministry of the Environment of Qatar map^21,22^. These potentiometric maps were produced from data collected through comprehensive surveys and field measurements. In the 2009 survey conducted by the Ministry of Environment, for instance, more than 8000 wells were identified and used in the analysis.

### Description of the geological formations and hydrogeology of the Qatar Peninsula

**Figure 5:**
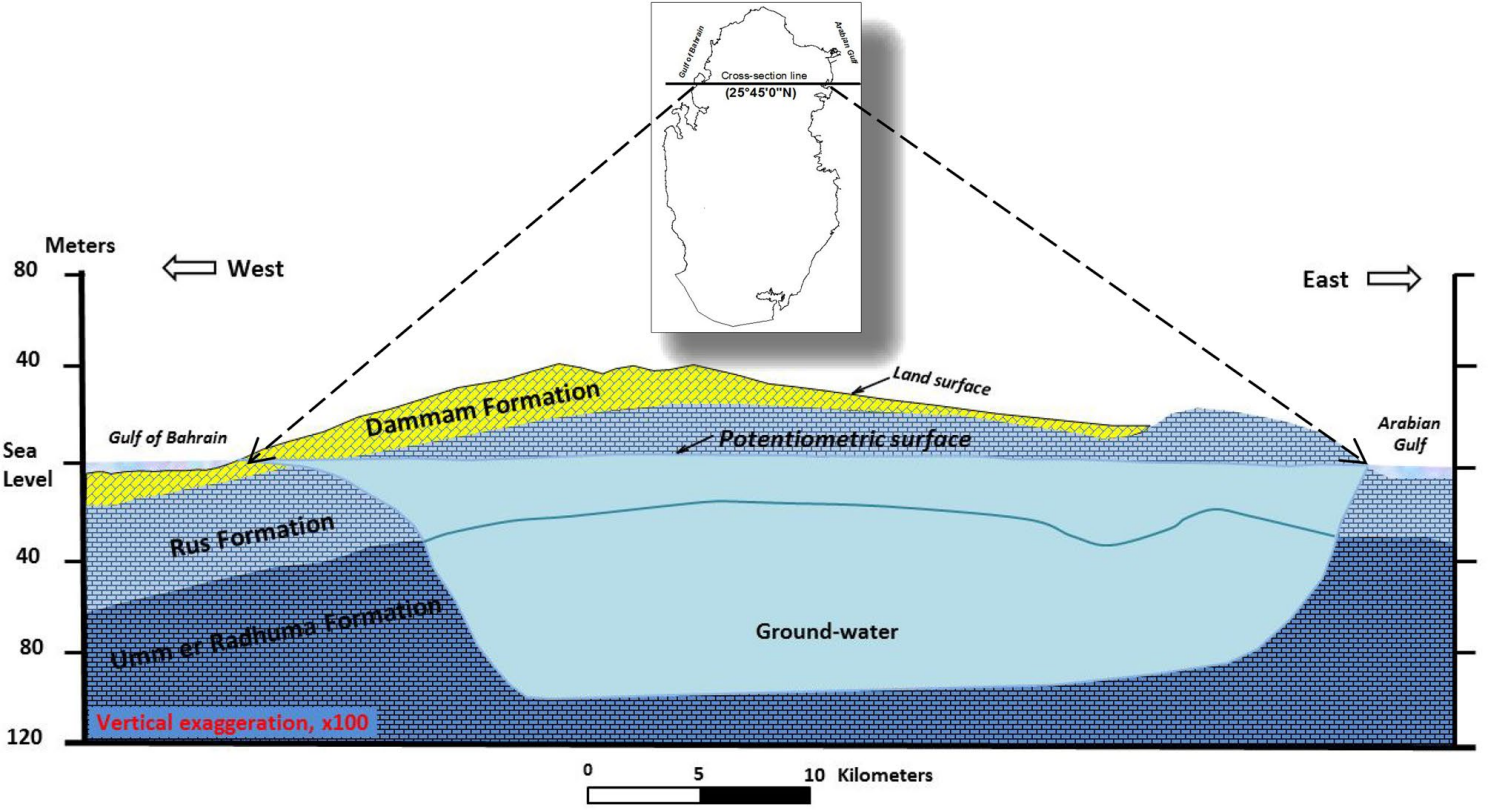
Hydrogeologic section in the north of Qatar. (Source: US Geological Survey, Open File. Report (76–540), http://pubs.usgs.gov/of/1976/0540/report.pdf)^23^.

The geological formations in Qatar are often described as “tertiary formation” as it is composed of three formations: Dammam; Rus; and Umm er Radhuma formations. This study focuses on the hydrologically connected Rus and Umm er Radhuma aquifers that form the “upper principal aquifer” since these are the main freshwater bearing formations in Qatar^23, P. 19^. The shallow surficial Dammam aquifer, found in some areas mostly within the coastal zones such as Doha basin, has limited storage capacity^24^. Figure 5 shows a hydrogeologic section along latitude 25° 45’^23^, depicting this Tertiary formation in northern Qatar where most of groundwater exist^23,25^.

Rus and Umm er Radhuma Formations in Qatar are unconfined to confined aquifer type^24^. In the last few- decades the Dammam and Rus and part of the upper part of Umm er Radhuma (the upper principal aquifer) have been heavily exploited for agricultural development locally and regionally, causing sharp drop in groundwater table and lateral and vertical shrinking of the freshwater lenses. 77% of the water abstraction in Qatar is from the Umm er Radhuma and Rus Aquifer and takes place in the north of the peninsula, whereas the remaining quantity is abstracted from the Rus aquifer found in the south of the country^24^.

**Figure 6:**
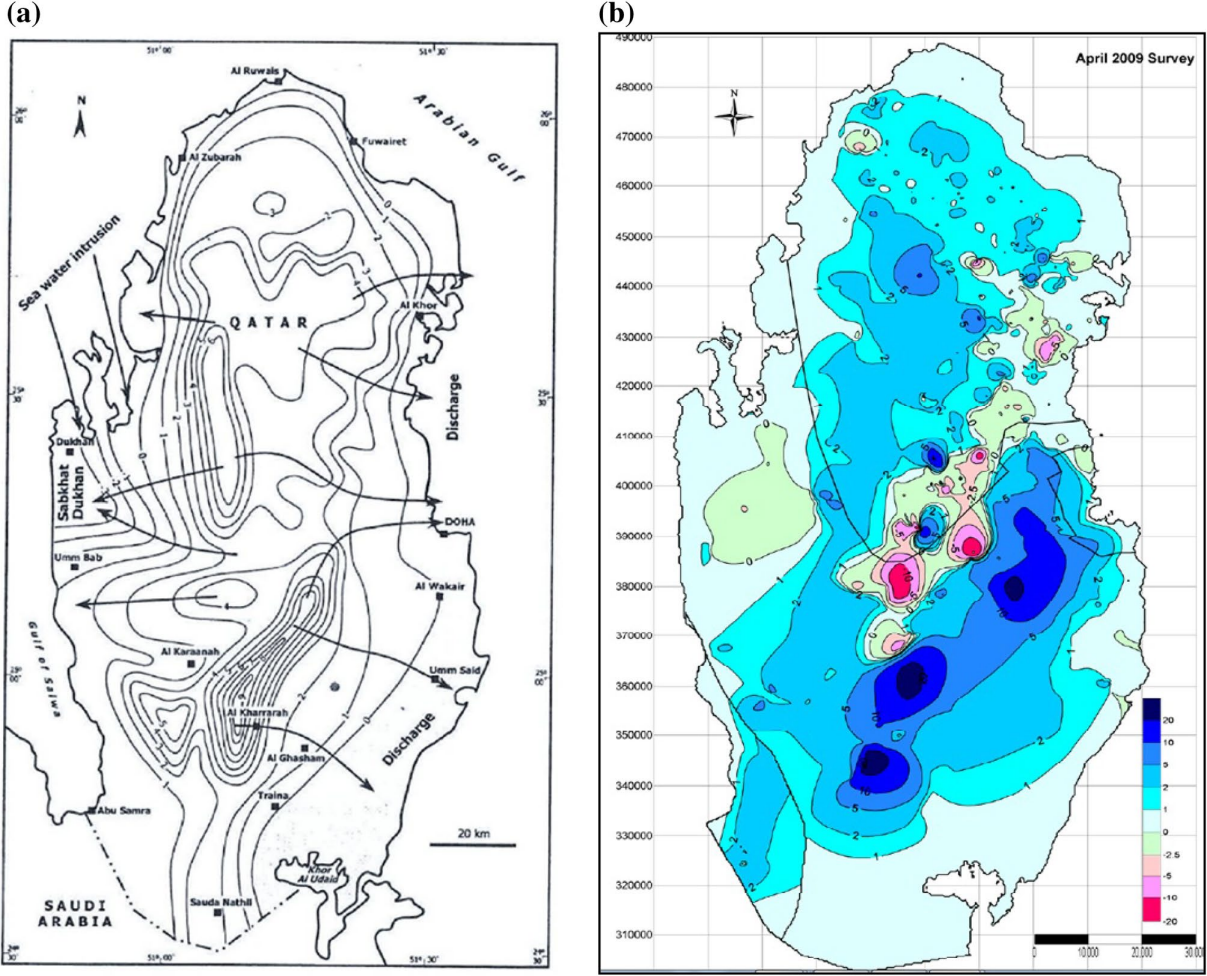
Potentiometric surface maps of the upper principal aquifer used in the study in the year. **(a)** 1980 (Source: Alsharhan et al. (2001), https://www.elsevier.com/books/hydrogeology-of-an-arid-region-the-arabian-gulf-and-adjoining-areas/alsharhan/978-0-444-50225-4)^22^. **(b)** 2009. (Source: Technical governmental report publically available, Ministry of Environment, the State of Qatar^21^.

The two maps used in this study represent potentiometric levels of upper principal aquifer in 1980 and 2009 respectively, are shown in Fig. 6a,b.

### Methodology

This section describes how the data have been prepared and processed and discusses the methods of analysis used to identify recharge sites/zones based on changes in the groundwater levels quantification. Data required for the estimation of the potential water volumes that could be used for artificial recharge is also discussed. ArcGIS software is used in this study, which is licensed to the Qatar Environment and Energy Research Institute (QEERI), Hamad Bin Khalifa University (HBKU), Qatar Foundation, Doha, the State of Qatar.

### Data preparation

The two potentiometric surface maps were digitized and converted from hardcopy to digital raster image format by scanning, before they can be manipulated and analyzed in the GIS system. These two raster images were then geo-referenced to the QNG 1995 national grid coordinate system in ArcGis system environment using Ground Control Points (GCP). The two raster maps were then converted to vector lines format and potentiometric values were assigned to their respective iso-height lines. The iso-heights were then converted to point data using the conversion tools in ArcGIS. Geo-statistics interpolation techniques of ArcGIS were then used to interpolate two continuous potentiometric surface maps (pixels) in order to be able to compare the pixel values of the same location of the two potentiometric maps.

### Surface interpolation

Spatial interpolation techniques in the GIS system were used to create two continuous potentiometric raster maps from the point data of 1980 and 2009, so that the two data sets could be compared and analyzed. ArcGIS offers two main groups of surface spatial interpolation techniques: deterministic; and geo-statistical. The former techniques are used for generating surfaces from measured points, based on either the extent of similarity or the degree of smoothing^26^. Geo-statistical techniques, on the other hand, utilize the statistical properties and spatial configuration of the measured points around the predicted location^26^, and quantify the spatial autocorrelation and configuration of these measured points.

Geo-statistical interpolation techniques were preferred over deterministic techniques, and that is based on the nature of the data available to this study. The *Ordinary Kriging* method of geo-statistical interpolation technique proved to provide accurate interpolation with minimum standard error for the data used in the study.

Two potentiometric raster surfaces, 1980 and 2009, were interpolated. The change in potentiometric levels between the two dates is obtained by subtracting of the 1980 potentiometric surface from the 2009 surface.

### Disclaimer

The manuscripts contents are solely the responsibility of the authors, and do not necessarily represent the official views of the Qatar University and the Environmental Science Center (ESC).

## Results and discussion

**Figure 7:**
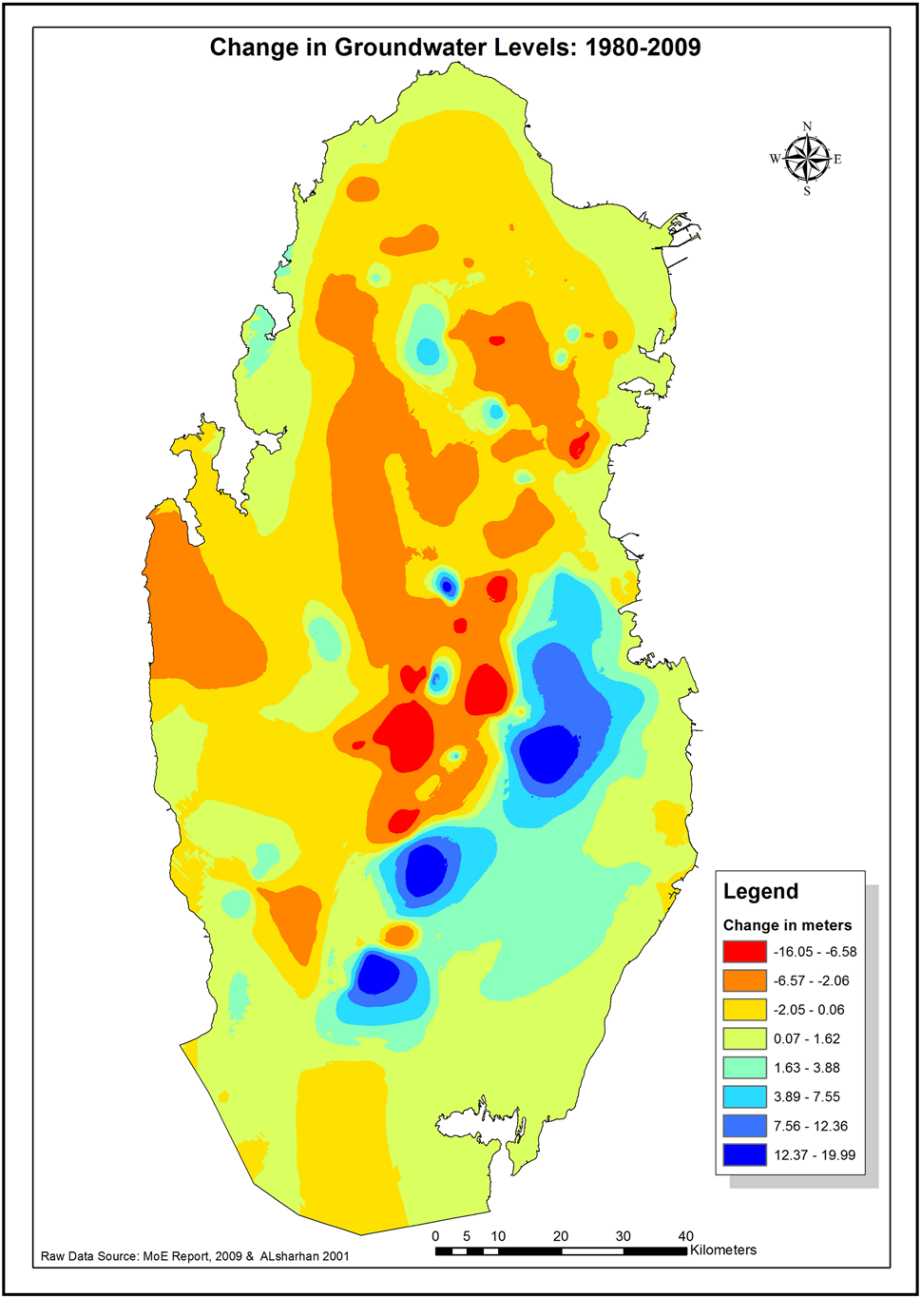
Change in groundwater level in Qatar between 1980 & 2009 by basin. Negative values indicate potential artificial recharge areas. −ve values represent drop in level, while + ve values represent increase in water level.

The 1980–2009 difference map is shown in Fig. 7 below. Negative values indicate decline/drop in the groundwater level (shown in red orange and yellow colors), whereas positive values indicate increase in water levels (shown in blue and other colors).

From the above figure, it is very clear that most of the decline/drop in the groundwater levels took place in the Northern basin, as consequence of over exploitation for irrigation. Areas with high decline in level have good potential for artificial recharge of aquifer and development.

**Figure 8:**
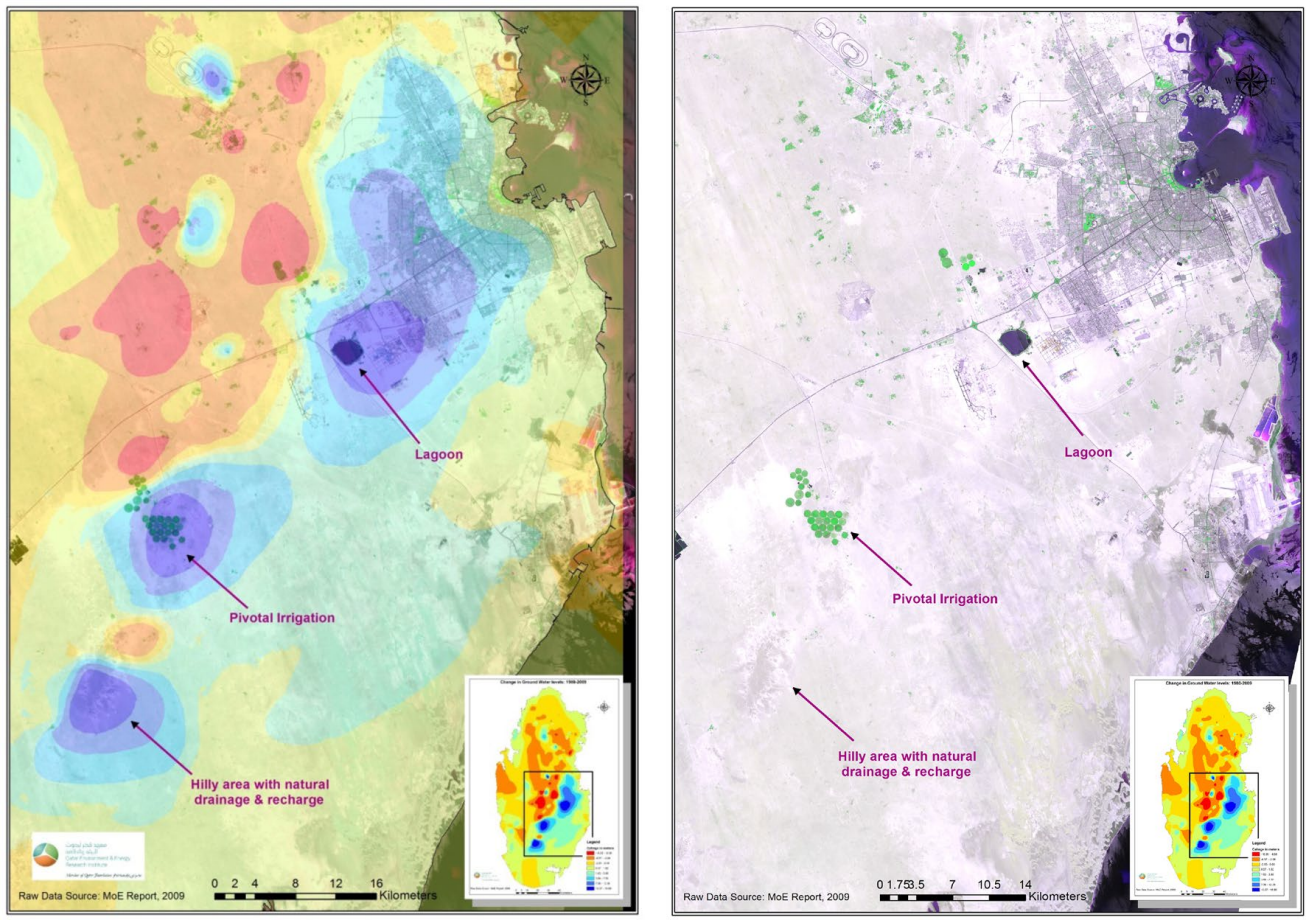
The resulted change in groundwater level map overlaid on Landsat 8 ETM + satellite image. Seepage from lagoons and pivotal irrigation systems result in aquifer recharge. Satellite Image processed by Authors using: (1) ENVI 4.8 by ITT Visual Information Solution: https://www.ittvis.com. (2) ArcGIS 10.4.1 software: Environmental Systems Research Institute (ESRI) at: https://www.esri.com.

The difference map was then overlaid on Landsat 8 satellite image from the Enhanced Thematic Mapper Plus (ETM +) sensor, of 2013, to help understand and better interpret the results. From the overlay, Fig. 8, the largest area with water level rise (blue color, in and around Doha City, is attributed to seepage from the unlined treated sewage water dumping lagoon at Abu Nakhla- south-west of Doha, as well as leakage from water distribution network in the city.

The other areas with level increase are the pivot-irrigation fields utilizing recycled treated water and the hilly area in south-west of Doha. These two areas act as a semi-natural aquifer recharge system, as can be seen in the north and the middle of the overlay.

**Figure 9:**
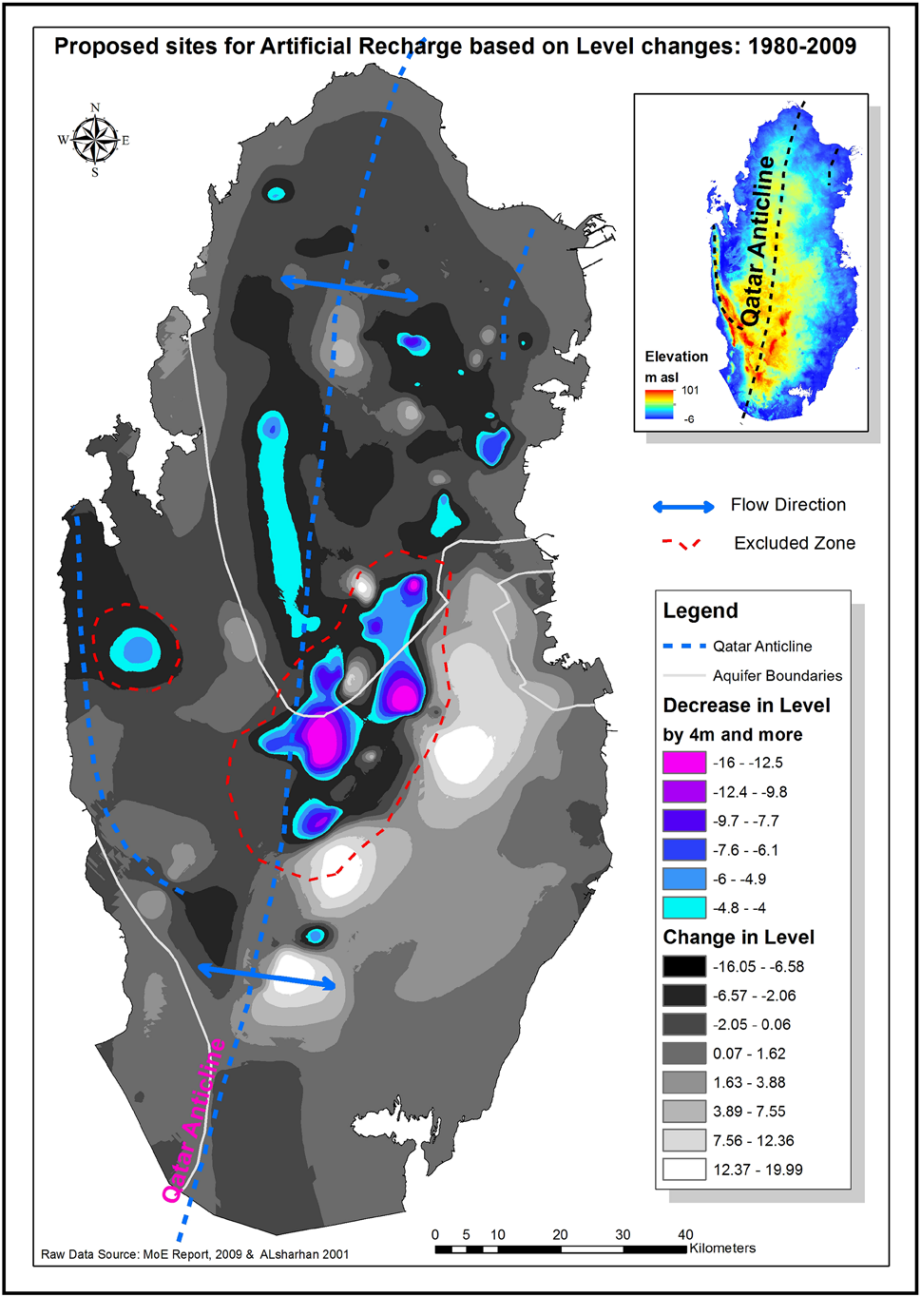
Areas with 4 m and more drop in the groundwater table.

Areas with groundwater level drops equal or greater than 4 m were considered as high potential zones for artificial recharge. The 4 m drop was chosen to insure enough storage space, in terms of layer thickness, in order to minimize risk of possible Land-Surface Deformation (LSD) as a result of artificial recharge^27^. Small LSD of few centimeters could have serious consequences for high-rise buildings in Doha City; such deformation causes cracks and damage to these buildings as the ground moves. Moreover, LSD also damages water and sewage network pipe-joints and fittings, exacerbating the water-leakage problem in Doha, resulting in the water-table rise in the city. ‘The rising water table in Doha has become a serious issue as it impedes the digging for construction foundations. Expensive de-watering procedures are required and special coating for the foundations is applied which add to the construction costs. Water leakage implies more desalinated water needs to be produced to substitute for the lost waters and increasing vulnerability to disasters in the Gulf’^27^. Figure 9 below shows the areas with water level drops of 4 m or more in groundwater level. The central areas marked with the dotted red line in the figure were excluded from the recharge plan, despite the high potential recharge volumes. These areas were excluded to avoid raising the groundwater levels in Doha City. ‘In Qatar, groundwater flows radially following the general trend of Qatar domal structure, outwards from recharge areas, centered over the higher Qatar Axis or anticline/homocline that plunges north- south with a surface expression of a broad shallow dome’^27^.

The water discharges into the adjacent low-lying land along the coast and the Gulf^23,24^. Artificially recharging these areas results in increased flow towards Doha, down the groundwater gradient. The highly treated municipal sewage effluent could be artificially recharged into the aquifer to recharge to sustain this non-renewable precious water source as reported by^28^.

**Figure 10:**
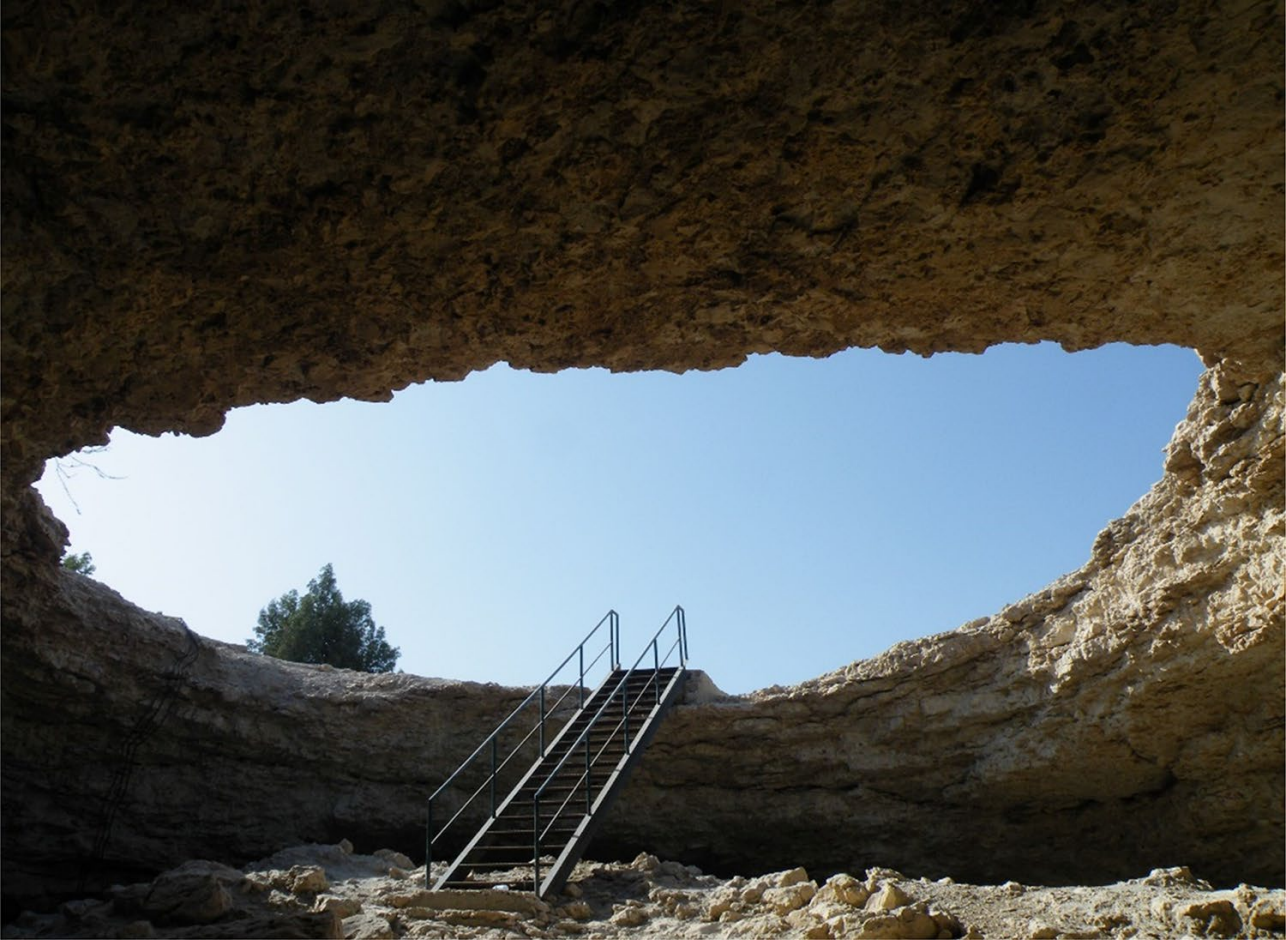
Photo of Dahl Al-Hamam sinkhole in the Qatar Peninsula. ( Source: taken by the second coauthor).

Another reason for excluding these central areas is that these areas have large number of sinkholes. These karst features are produced by the dissolution of subsurface gypsum beds during humid and wet periods in the Pliocene and Pleistocene period. Refilling these gypsum formations with freshwater is very likely to cause re-dissolution, and hence the collapse of ground surface. The Dahl Al-Hamam sinkhole is an example of these sinkholes shown in Fig. 10 below.

### Potential water-storage volume calculation

**Figure 11:**
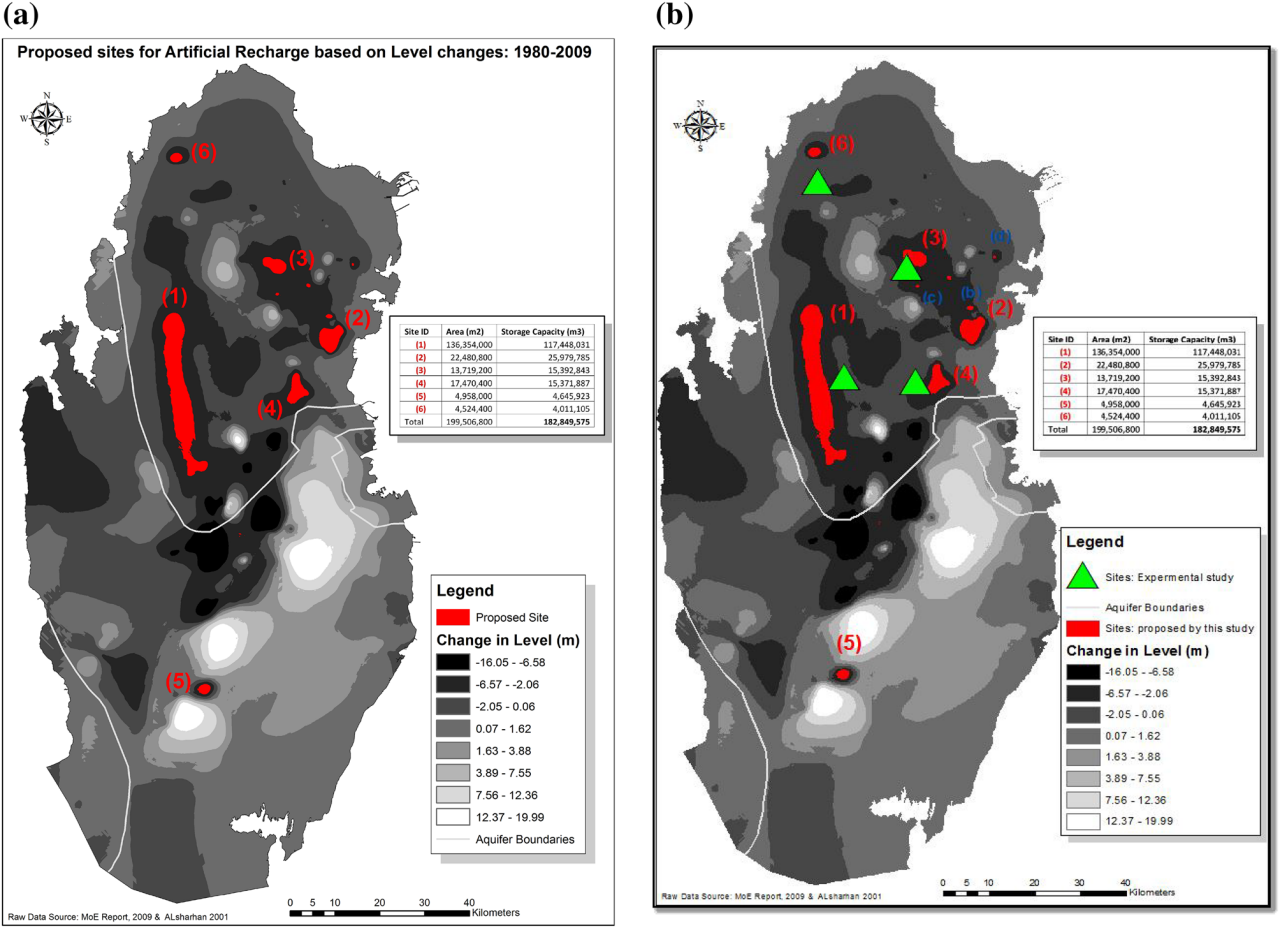
**(a)** Proposed sites for potential water-recharge sites (in red color). (**b)** Proximity of experimental sites (green tringles) to the proposed zones (red polygons) identified in this study.

To calculate how much water could potentially be recharged in upper principal aquifer in the selected areas identified in Fig. 9 above. Zones with drop in water levels equal or greater than 4 m are considered as a high storage capacity potential sites. First a mask for these zones was created using the ‘Change in Groundwater levels’ map shown in Fig. 8. The volume of change in water levels is calculated using Cut/Fill tool in ArcGIS software. Since soil’s water holding capacity is determined by soil porosity, soil porosity for the major soil formation in the area (Limestone) was used to determine the actual water volume potentials. Limestone porosity factor of 20% was used in this study^29–31^. The actual water volume potential is calculated by multiplying the calculated volume by porosity factor. Results show that there are major six sites with high storage potentials which totals to 182.9 mcm of water, see Fig. 11a below. Moreover, four minor/secondary sites allocated between sites 2 and 3, with storage potentials of 2 mcm. These secondary sites could be used for testing purposes since they are small and hence easier to monitor.

### Identification of the optimum recharge sites

The outcomes of this study identify six major areas/zones which are likely to have high artificial recharge potentials, presented in Fig. 11b below. These sites are characterized by geological formations composed of fissured and fractured carbonate depositional facies of the Tertiary Period, which is considered as favorable layers for groundwater recharge and storage. In addition to that most of these sites are situated within the northern groundwater province, the bearing layers of the freshwater lenses, which is being abstracted heavily for irrigation in the active farms in the State of Qatar.

To verify the results and suitability of the proposed sites, experimental fieldwork is required. However, due to lack of time and resources, results from an extensive existing experimental fieldwork of Streetly et al. study (conducted in 1992) in northern Qatar, published in 1998^31^, have been used as ground-truthing validate this study.

Streetly et al.’s study is based on field testing and modeling of the principal upper aquifer (Rus and Umm er Radhuma aquifers at four sites^31^ situated within the depositional carbonate lithofacies aquifer. These four sites were found to be in relatively close proximity to the zones identified in this study, therefore representative of these zones, as shown in Fig. 11b, where the four sampling sites are depicted as green triangles in the map. This experimental work is based on step tests, constant rate tests, tracer recharge/re-abstraction, solute transport, hydrogeological modelling, determination of hydrogeological and geophysical parameters of aquifers, and soil properties. The Streetly et al.’s measurements identified these sites as optimum recharge locations, which endorses findings in this study.

In 2018 water production from desalination in Qatar reached 2.18 mcm/day^6,32^, (see Fig. 1). Accordingly, the 182.8 mcm potential storage would be enough for 3 months, if normal water consumption (business as usual) is assumed. However, if emergency measures were in place in case of water shortage, by water demand-management this storage would last longer. For instance, if the per capita daily consumption is equated to the per capita global average daily consumption in case of emergency, this period could be extended to over 1 year.

## Conclusion

Qatar has very limited strategic water reserve enough for only seven days. The country relies heavily on desalinated water from the Gulf and the groundwater is highly depleted. This reliance on desalination makes Qatar very vulnerable to potential natural and man-made disasters in the Gulf that could prevent water intake by the desalination plants.

Artificial recharge of groundwater aquifers, by freshwater, is one strategic measure proposed to increase the strategic water reserve of the country. The goal of this framework is to restore and regain the 1980s groundwater levels. To replenish these aquifers efficiently, a key question that determines success or failure of the plan is: where to recharge, and how to determine the optimum recharge sites?

It is suggested in this study that old natural storages that have experienced considerable falls and drop in water levels are optimum locations for water recharge since these storages were chosen by nature hundreds of years ago- based on the “natural flow” of groundwater. Working with nature is the optimum option for recharge. GIS-based approach and potentiometric surfaces were used to identify changes of groundwater levels to identify these optimum recharge sites/zones.

Experimental fieldwork based on hydrological, hydrogeological and geotechnical investigations validate and confirms the feasibility of the recharge sites identified by this study.

It is recommended that interferometry surveys with polarimetric RADAR, be conducted before and after the artificial recharge in order to monitor possible land surface deformation.

This study also proved that using GIS and potentiometric surface data could be cost and time-effective for preliminary identification of optimum recharge sites to pave the way for limited experimental fieldwork. Moreover, the proposed methodology and framework would achieve water security issues successfully and could be applicable in similar arid environments.
